# PropaNet: Time-Varying Condition-Specific Transcriptional Network Construction by Network Propagation

**DOI:** 10.3389/fpls.2019.00698

**Published:** 2019-06-14

**Authors:** Hongryul Ahn, Kyuri Jo, Dabin Jeong, Minwoo Pak, Jihye Hur, Woosuk Jung, Sun Kim

**Affiliations:** ^1^Bioinformatics Institute, Seoul National University, Seoul, South Korea; ^2^Interdisciplinary Program in Bioinformatics, Seoul National University, Seoul, South Korea; ^3^Department of Computer Science and Engineering, Seoul National University, Seoul, South Korea; ^4^Department of Crop Science, Konkuk University, Seoul, South Korea

**Keywords:** transcription factor, gene regulatory network inference, time-varying, plant stress, influence maximization, network propagation

## Abstract

Transcription factor (TF) has a significant influence on the state of a cell by regulating multiple down-stream genes. Thus, experimental and computational biologists have made great efforts to construct TF gene networks for regulatory interactions between TFs and their target genes. Now, an important research question is how to utilize TF networks to investigate the response of a plant to stress at the transcription control level using time-series transcriptome data. In this article, we present a new computational network, PropaNet, to investigate dynamics of TF networks from time-series transcriptome data using two state-of-the-art network analysis techniques, influence maximization and network propagation. PropaNet uses the influence maximization technique to produce a ranked list of TFs, in the order of TF that explains differentially expressed genes (DEGs) better at each time point. Then, a network propagation technique is used to select a group of TFs that explains DEGs best as a whole. For the analysis of Arabidopsis time series datasets from AtGenExpress, we used PlantRegMap as a template TF network and performed PropaNet analysis to investigate transcriptional dynamics of Arabidopsis under cold and heat stress. The time varying TF networks showed that Arabidopsis responded to cold and heat stress quite differently. For cold stress, bHLH and bZIP type TFs were the first responding TFs and the cold signal influenced histone variants, various genes involved in cell architecture, osmosis and restructuring of cells. However, the consequences of plants under heat stress were up-regulation of genes related to accelerating differentiation and starting re-differentiation. In terms of energy metabolism, plants under heat stress show elevated metabolic process and resulting in an exhausted status. We believe that PropaNet will be useful for the construction of condition-specific time-varying TF network for time-series data analysis in response to stress. PropaNet is available at http://biohealth.snu.ac.kr/software/PropaNet.

## 1. Introduction

A transcription factor (TF) is a protein that regulates expression levels of a target gene (TG) by binding to a specific DNA sequence on the promoter regions of target genes (Latchman, 1997). TFs activate or repress target genes, contributing to expression of genes at the right time and in the right amount throughout the life of the cell and the organism. Groups of TFs function in a coordinated fashion to direct various biological mechanisms such as development (Lobe, 1992), signal transduction (Pawson, 1993), response to environmental change (Chen et al., 2002), and regulation of cell cycle (Meyyappan et al., 1996).

When a plant is exposed to stress, TFs that are activated in response to stress rapidly propagate stress signals to other genes in the cell by regulating multiple downstream genes (Zhu, 2016). In recent genetic engineering experiments of plants, over-expression of specific transcription factors enhanced resistance to stress. For example, over-expression of TFs such as OsAP37 (Oh et al., 2009), OsNAC9 (Redillas et al., 2012), OsNAC10 (Jeong et al., 2010), MYB96 (Seo et al., 2011), OsbZIP12 (Joo et al., 2014), and OsbZIP23 (Karaba et al., 2007) induced drought resistance phenotype in rice and Arabidopsis. Thus, the TF gene regulatory network (or TF network) should be considered as an essential source of information when detecting stress response signaling genes.

However, many computational methods for investigating the stress response genes of plants do not utilize TF networks. Algorithms such as EDISA (Supper et al., 2007), a two-step 3D clustering algorithm (Supper et al., 2007), OPTricluster (Tchagang et al., 2012), and TimesVector (Jung et al., 2017) perform 3D clustering which takes advantage of gene, time and phenotype information, yet, they do not take account of TF network. The primary goal of the methods is to detect a group of genes that show same expression patterns in all phenotypes (coherent response) or show same patterns except in one phenotype (single response) or show different patterns for different phenotypes (independent response). All of these methods successfully detected important gene clusters, but it was not sufficient to detect regulatory relationship between clustered genes without prior knowledge of TF-TG network.

Construction of transcriptional networks has been extensively investigated in both computational biology and experimental biology. In this paper, the TF gene regulatory network (or TF network) refers to a graph-based representation that contains regulatory relationships between TFs and their target genes. Constructing a TF network is a very difficult task since the number of possible TF-TG relationships to be considered for the construction of a TF network is over 30 million (1,500 TF × 20,000 TG); There are 20,418, 22,619 and 27,665 protein-coding genes (i.e., TGs) in human, mouse, and Arabidopsis species, respectively, according to the ENSEMBL (Zerbino et al., 2017). However, a few number of TF network databases have been produced, thanks to the large scale efforts of research communities, e.g., PlantTFDB (Jin et al., 2016), HumanTFDB and AnimalTFDB (Hu et al., 2018).

There are three different strategies for building TF networks. First, the most fundamental approach would be to collect TF-TG relationships from literatures. Biologists conducted various types of biological experiments such as over-expression, knock-down or knock-out of genes, in-depth expression level profiling, for elucidating TF-TG relationships. The number of TF-TG relations in the literature-based databases is relatively small, but the information is reliable. TRRUST (Han et al., 2017) contains 8,444 and 6,552 TF-TG interactions for 800 and 828 TFs for human and mouse species, respectively. In ATRM (Jin et al., 2015), 1,431 TF-TG interactions for 324 TFs are curated for Arabidopsis. HTRIdb (Bovolenta et al., 2012), PAZAR (Portales-Casamar et al., 2007), TFactS (Essaghir et al., 2010), TRED (Zhao et al., 2005) and TFe (Yusuf et al., 2012) are examples of the literature-based TF network construction.

The second approach is based on computational predictions from a large collection of gene expression datasets that are measured using high-throughput technologies such as microarray (Schena et al., 1995) and RNA sequencing (Mortazavi et al., 2008). ARACNe (Margolin et al., 2006) uses an information theoretic framework based on the data processing inequality theorem. It was successfully used for the reconstruction of context-specific transcriptional networks in multiple tissue types (Lefebvre et al., 2010). Then, many methods were developed for the construction of TF networks: GENIE3 (Huynh-Thu et al., 2010), NARROMI (Zhang et al., 2012), Ennet (Sławek and Arodź, 2013), and Wisdom of crowds (Marbach et al., 2012). Following these successes, the DREAM (Dialogue for Reverse Engineering Assessments and Methods) challenge competition was initiated. Yip's method (Yip et al., 2010) (for DREAM3) and GENIE3 (Huynh-Thu et al., 2010) (for DREAM4 and DREAM5) won the competitions. These approaches were further developed to investigate dynamics of TF networks. Various gene regulatory network (GRN) inference methods using time-course data have been developed with model-based and model-free approaches. Model-based methods formulate the expression of a target gene as a function of its regulators. The representative model-based methods, such as ScanBMA (Young et al., 2014), Inferelator (Bonneau et al., 2006), BGRMI (Iglesias-Martinez et al., 2016), Fused-Lasso (Omranian et al., 2016). BGRMI (Iglesias-Martinez et al., 2016), use ridge regression, LASSO and Bayesian Model Averaging (BMA) techniques. Model-free methods compute the degree of regulation based on model-free and time-lag regression. TD-ARACNE (Zoppoli et al., 2010) used mutual information to measure time-delayed dependency between two genes. DyeGENIE3 (Huynh-Thu and Geurts, 2018) and BTNET (Park et al., 2018b) used tree-based ensemble regression methods.

The third approach uses experimental data that measure affinity of TFs. The discovery that formaldehyde can crosslink histones to DNA (Brutlag et al., 1969) initiated Chromatin Immunoprecipitation (ChIP) assay that utilized crosslinked complexes and analysis of the associated DNA (Solomon and Varshavsky, 1985). Formaldehyde produces both protein-nucleic acid and protein-protein crosslinks *in vivo* by a reaction with amino and imino groups of amino acids and of DNA (Orlando, 2000). ChIP assays performed with crosslinking have made it possible to identify interactions that would not withstand the isolation procedure without crosslinking (Hoffman et al., 2015). ChIP assay has been ubiquitous in a multiple variations, one of which is ChIP-on-chip that combines ChIP with DNA microarray (Ren et al., 2000). Several studies identified binding sites for TFs by ChIP-on-chip in plants including Arabidopsis (Thibaud-Nissen et al., 2006). ChIP sequencing (ChIP-seq) technology was developed independently by three research groups in 2007 (Barski et al., 2007; Johnson et al., 2007; Mikkelsen et al., 2007) and it has been used to identify genomic regions that TF binds to, also known as, transcription factor binding sites (TFBSs). It crosslinks DNA and associated TFs, shears DNA-TF complexes into 500 bp DNA fragments by sonication or nuclease digestion, immunoprecipitates the targeted TF complexes using an appropriate protein-specific antibody, and then determines the sequence of the DNA fragments. With ChIP-seq and several other variants of immunoprecipitation assay such as ChIP-chip (Ren et al., 2000), ChIP-exo (Rhee and Pugh, 2011), ChIA-PET (Fullwood and Ruan, 2009), a number of ChIP-seq-like datasets for different species, tissues and cell lines have been generated and are freely available in databases such as Gene Expression Omnibus (GEO) (Barrett et al., 2013), Sequence Read Archive (SRA) (Kodama et al., 2011) and ENCODE (Landt et al., 2012). We can locate a binding motif sequence of a TF by processing ChIP-seq dataset and predict the target genes by searching the binding motif sequence on the promoter region of target genes. Now, some of the databases are providing TF-TG relationships by predicting binding sites for the collective TFs: TRANSFAC (Matys et al., 2006) a well-known commercial database; ENCODE (Landt et al., 2012), JASPAR (Khan et al., 2017) and ChIP-Atlas (Oki et al., 2018) for model organisms; GTRD (Yevshin et al., 2016), ChIPBase (Yang et al., 2012), Cistrome (Zheng et al., 2018b) and Factorbook (Wang et al., 2012) for human and mouse species; PlantRegMap (Jin et al., 2016) for plant species.

## 2. Motivation

Investigating time-varying dynamics of TF network upon abiotic stress is the main research question. We can use a template network from existing TF networks that are surveyed in the previous section. A biological experiment can be designed to investigate how a plant responds to stress over time by measuring transcriptome data at different time points under stress. Then, cell's response at the transcriptome level can be easily detected by measuring differentially expressed genes (DEGs), control vs. under stress, at each time point. By constructing TF networks that include DEGs and TFs, we can gain insight into how the responses of the TFs differ in gene expression levels under each stress, i.e., DEGs. There are two major issues with this approach.

1. Contributions of TF to DEGs differ for different TFs. It is not enough to consider only TFs that show significant expression changes during stress. It is known that TFs that show little change in expression levels function in response to stress as much as those that show large change in expression levels. In other words, the amount of change in expression level of a TF is not necessarily proportional to the significance of its role in the response to the stress (Ehlting et al., 2008; Larkindale and Vierling, 2008). In addition, determining major regulators is not trivial since a TF can target other TFs, forming multi-layered relationships to reach DEGs. How do we know which TFs are the major regulators?
2. Since there are many TFs and DEGs at each time point, TF networks are huge in size, which are too big to be interpreted. How can we construct small but informative TF networks at each time point so that we can interpret dynamics of TF networks?

Unfortunately, there is few computational methods to achieve this goal of investigating the dynamics of TF networks from time-series gene expression data. There are some interesting prior works, but they are not designed for answering this research question. DREM (Schulz et al., 2012) is a pioneering method for the identification of regulatory TFs from the time-series gene expression data analysis. It first partitions genes into gene clusters, then defines branch time points where gene expression patterns are diverging, and then investigates the effect of TFs to the downstream genes utilizing known TF-TG relationships to detect regulatory TFs for gene clusters. However, DREM uses only direct TF-TG information. Therefore, it cannot consider the effects of interactions between TFs that might cause indirect influence on differential expression of genes. TimeTP (Jo et al., 2016) is another method for detecting major TFs using Influence Maximization (IM), one of the widely used analysis techniques for social network analysis. TimeTP produces a list of TFs having influence on pathway genes, but it is not designed to investigate time-varying dynamics of TF networks. There are network construction methods that utilized the literature information. For example, Wanke et al. (2010b) constructed a network of MAPK (mitogen-activated protein kinase) signaling pathway including receptors, second messengers and TFs from known interactions in literature. In general, literature based network construction requires quite efforts involving manual curation. Thus, these methods are not suitable for large scale network construction involving multiple pathways.

In this article, we propose PropaNet to investigate dynamics of TF networks from time-series transcriptome data through network simulation analysis that mimics the regulatory mechanism of TF. PropaNet uses state-of-the-art network analysis technologies, influence maximization and network propagation techniques, to perform a two-stage simulation analysis as below.

1. An influence maximization technique is used to produce a ranked list of TFs at each time point, in the order of TFs that have influence on a larger number of DEGs.
2. A network propagation technique is used to select a group of TFs that explains DEGs best as a whole. The process is done by iterating a network propagation simulation by adding a TF at a time, going down the list of TFs determined by the influence maximization technique.

We used PropaNet to interpret time-series analysis transcriptome data under cold and heat stress in Arabidopsis. From the analysis, we successfully identified major TFs and constructed TF networks at each time point. “A major TF” indicates a TF that has large direct or indirect influence on DEGs (estimated by influence maximization) and propagates more influence on more significant DEGs (estimated by network propagation). Performance of PropaNet was compared with simple correlation-based methods and other methods for time series analysis using clustering and network information. PropaNet showed better performance in finding cold- and heat-specific transcription factors and their target genes by incorporating network information and its novel strategy for selecting major regulatory TFs.

## 3. Methods

### 3.1. Problem Definition of PropaNet

The PropaNet analysis takes three types of input data: time-series gene expression data *EX* that are measured at multiple time-points, a template TF network *G* and a set of target genes *TGset*. PropaNet detects major TFs that particularly target *TGset* defined by users. *TGset* can range from a small set of pathway genes to whole DEGs of the gene expression data. The goal of the PropaNet analysis is to elucidate time-varying networks of major TFs and their target genes at each time point by the network-based analysis on the template TF network. Terminologies used in this paper are defined as follows:

*Definition 3.1*. Let *EX*, *G* and *TGset* be time-series gene expression data, TF network and a set of targeted genes, respectively. *EX* is a set of gene expression values *e*_*i,j,k*_ of gene *i* measured at time point *j* = 0, …, *T* from replicate *k* = 1, …, *K*. A set of differentially expressed genes for time point *j* = 1, …, *T* compared to the initial time point *j* = 0 is defined as *DEGset*_*j*_. From the template network *G*, a time-specific network *G*_*j*_ is generated using a gene set *V*_*j*_ = (*TGset* ∩ *DEGset*_*j*_) ∪ *TFset*. A time-specific network *G*_*j*_ is defined as *G*_*j*_ = (*V*_*j*_, *E*_*j*_) with nodes *V*_*j*_ (TFs and TGs) and edges *E*_*j*_ (TF-TF and TF-TG pairs). For each node and edge, a weight of a node *p* : *V* ↦ ℜ (a map of node to weight) and a weight of an edge *w* : *E* ↦ ℜ (a map of edge to weight) exist. Differential expression levels *d*_*i, j*_ and correlation coefficient $c_{i,i^{\prime }}$ that are defined below is assigned to *p* and *w*, respectively.

- *D*_*j*_ is a set of differential expression levels at time *j*, the element of which *d*_*i,j*_ is the differential expression level of a gene *i* at the time point *j* = 1, …, *T* with respect to the initial time point *j* = 0. That is, *d*_*i,j*_ is calculated from comparing two sets of expression values {*e*_*i,j*,1_, …, *e*_*i,j,k*_} and {*e*_*i*,0,1_, …, *e*_*i*,0,*k*_} by an existing DEG detection algorithm such as limma (Ritchie et al., 2015) or DESeq2 (Anders and Huber, 2010). *d*_*i,j*_ can be Z-scores from limma or log2 fold change from DESeq2.
- $c_{i,i^{\prime }}$ is a Pearson's correlation coefficient (PCC) between gene *i* and *i*′ computed using two sets of expression values {*e*_*i,j,k*_} and $\left\{e_{i^{\prime },j,k}\right\}$ where *j* = 1, …, *T, k* = 1, …, *K*.
- *DEGset*_*j*_ is a set of differentially expressed genes (DEGs) at time point *j* where a DEG is defined as a gene with p-value(*d*_*i,j*_) < 0.05.
- *MTFset*_*j*_ is a minimal set of major TFs that explains the change of expression levels of *DEGset*_*j*_ at time point *j*.
- *MTFnet*_*j*_ is a time-varying TF network at time point *j* that shows the regulatory system explaining how *MTFset*_*j*_ controls *DEGset*_*j*_.

**Figure 1:**
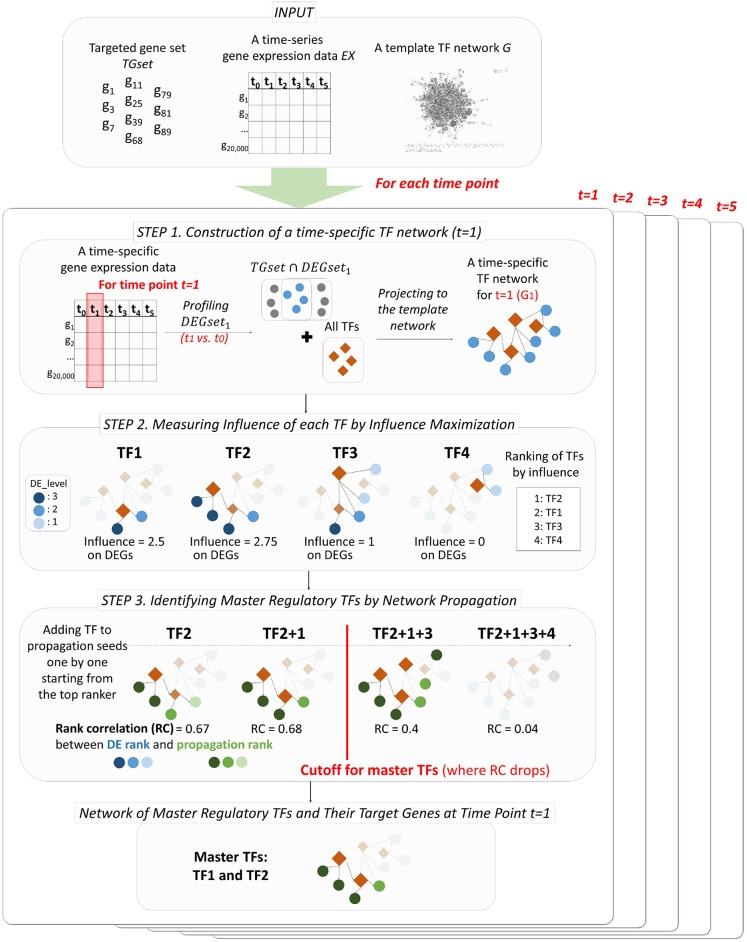
Workflow of PropaNet. The PropaNet analysis takes three types of input data: time-series gene expression data that are measured at multiple time-points, a template TF network and a set of targeted genes. The goal of the PropaNet analysis is to elucidate time-varying networks at each time point. It uses the influence maximization technique to produce a ranked list of TFs at each time point, in the order of TF that explains DEGs better. Then, a network propagation technique is used to select a group of TFs that explains DEGs best as a whole. The process is done by iterating a network propagation simulation by adding a TF at a time, going down the list of TFs determined by the influence maximization technique.

PropaNet outputs a set of major regulatory TFs *MTFset*_*j*_ and their time-varying network including target genes *MTFnet*_*j*_ for each time point *j* = 1, …, *T*. PropaNet operates in three steps as below and the process is visualized in Figure 1.

- Step 1. Instantiation of time-specific TF networks from a template network. A time-specific network consists of DEG and TFs at each time point.
- Step 2. Time-specific measurement of the influence of each TF by influence maximization. TFs in the network are ranked along with their influence on DEGs via the network topology (including non-direct targets).
- Step 3. Identification of time-specific major regulatory TFs by network propagation. The TF set is constructed by adding a TF at a time, following down the ranked list of TFs.

### 3.2. Step 1. Instantiation of Time-Specific TF Networks

The first step of PropaNet is to construct a time-specific networks *G*_*j*_ for each time point *j* = 1, …, *T* by mapping TFs and a intersected gene set of user-defined target genes and DEGs to a template network (i.e., a gene set *V*_*j*_ = (*TGset* ∩ *DEGset*_*j*_) ∪ *TFset* for time *j*) (Figure 1). DEGs are determined by comparing gene expressions at each time point *j* = 1, …, *T* with time *j* = 0 (i.e., 0 h after stress). The inputs of PropaNet (a template TF network and DEG profiles) are designed to be user-determined.

### 3.3. Step 2. Time-Specific Measurement of the Influence of Each TFs by Influence Maximization

The goal of this step is to rank TFs in the order of influence to *TGset* ∩ *DEGset*_*j*_ at each time point *j*. Influence maximization (IM) is an algorithmic technique used in network influence analysis to select a set of seed nodes to maximize the spread of influence (the expected number of influenced nodes) from a given network (Li et al., 2018). Labeled influence maximization (Li et al., 2011) is a modified version of IM, applied only to a set of pre-selected nodes. TimeTP (Jo et al., 2016) used the labeled influence maximization algorithm to determine TFs that regulates the perturbed sub-pathways. We used a modified version of the labeled influence maximization algorithm to rank TFs in terms of their influence to *TGset* ∩ *DEGset*_*j*_ at time point *j*.

We provide more detailed explanation of the labeled influence maximization algorithm of PropaNet (Algorithm 1). Input of the algorithm for time *j* is (*G*_*j*_ = (*V*_*j*_, *E*_*j*_), *TFset, D*_*j*_) to measure the influence of each TF (∈ *TFset*) to the targeted genes (∈ *V*_*j*_\*TFset*) on the time-specific TF network *G*_*j*_. IM algorithm first initializes the weights of node, *DE*(*s*), as the absolute differential expression level *d*_*s,j*_ for all *s* ∈ *V* and the influence of TF, *IL*(*t*), as 0 for *t* ∈ *TFset*. Then, it generates sub-graph *G*′ from *G* by selecting edges with a probability of 1 − *p* for each edge (line 4), where *p* is the weight of the edge in the original graph *G*. Then, the influence *IL*(*t*) increases by the $\sum DE\left(s^{\prime }\right)/|AllReachableNodes_{G^{\prime }}\left(t\right)|$ for $s^{\prime }\in AllReachableNodes_{G^{\prime }}\left(t\right)\backslash TFset$ where $AllReachableNodes_{G^{\prime }}\left(t\right)$ is nodes that the TF can reach in the generated sub-graph *G*′. After repeating the above procedure for *Round* times, the algorithm produces the final output *IL* of all TFs at the time point *j*.

**Algorithm 1:** Influence Maximization on TF-TG network (*G, TFset,D*)

1:	Let		
		*G* = (*V*, *E*) *TFset* *D* = *d_*i,j*_*	: a TF network defined on the nodes *V* : a set of TFs : values of differential expression of gene *i* at time point *j*
2:	Initialize		
		*DE(s)* = *abs*(*d_s,j_*), for all *s* ∊ *V* *IL*(*t*) = 0, for all *t* ∊ *TFset* *Round* = 1000	: an absolute differential expression of a genes : an influence level of TF*t* : the number of iteration
3:	**for** *i* ← 1, …, *Round* **do**		
4:	Derive *G*′ by removing each edge from *G* according to the probability 1 − *p* where *p* is the weight of the edge in the original graph *G*.		
5:	**for each** node *t* ∈ *TFset* **do**		
6:	$IL(t)\leftarrow IL(t)+\sum_{s\in AllReachableNodes_{G^{\prime }}(t)\setminus TFset}\frac{DE(s)}{\left|AllReachableNodes_{G^{\prime }}(t)\right|}$		
7:	**end for**		
8:	**end for**		
9:	Normalize *IL*(*t*) = *IL*(*t*)/*Round*, for all *t* ∈ *TFset*.		
10:	**return** *IL*		

### 3.4. Step 3. Time-Specific Identification of Major Regulatory TFs by Network Propagation

Network propagation is a graph-based analytic paradigm that propagates information of a node to nearby nodes through the edges at each iteration step. This process is repeated for a fixed number of steps or until convergence. Since the value of a node influences not only the values of its direct network neighbors but also those of its distant neighbors, network propagation is known to perform better than direct neighbor search methods and shortest path search methods for a problem of prioritizing genes that are associated with seed genes (Cowen et al., 2017).

Network propagation is mathematically equivalent to random walks on a graph. We can think of *p*_0_(*v*) as an amount of information of a node *v* at the beginning of iteration 0. At each iteration *k*, the amount of information at each node *v* is influenced by the sum of the information at the neighboring (adjacent) nodes *N*(*v*) at iteration *k* − 1, in proportion to the weights on the corresponding edges, according to the following equation:

(1) $$\begin{array}{l} p_{k}\left(v\right)=\underset{u\in N\left(v\right)}{\sum }p_{k-1}\left(u\right)w\left(u,v\right), \end{array}$$

where *w*(*u, v*) is the weight of the edges (*u, v*) in the input network. The propagation process described in Equation 1 can be written in matrix notation as follows:

(2) $$\begin{array}{l} p_{k}=Wp_{k-1}, \end{array}$$

where *W* is a normalized version of the adjacency matrix of the input network. Another version of the propagation process is the random walk with restart (RWR). RWR performs the random walk and restarts at a rate of α:

(3) $$\begin{array}{l} p_{k}=\alpha p_{0}+\left(1-\alpha \right)Wp_{k-1}, \end{array}$$

where the parameter α is thought of the trade-off between prior information (restart) and network smoothing (random walk). After *k* iterations, the values in the resulting vector *p*_*k*_(*v*) give us a ranking of each node *v* that diffused from the initial value of seed nodes.

PropaNet simulates the TF-centered regulation process based on the network propagation. Each step of network propagation outputs ranked list of nodes in the network. The objective function, or the stopping criteria, is to find a ranked list of genes that are most similar to the ranked list of DEGs in terms of their *p*-values. This can be easily determined by computing Spearman's rank correlation coefficient (SCC) between the two ranked lists. More formally, the simulation is evaluated by the comparison of the ranking between the differential expression of observation *DE*(*v*) and the inferred expression of network propagation *IP*(*v*). This simulation is independently processed for each time point *j*. It first initializes the information of nodes, *IP*(*v*) as 0 for *v* ∈ *V*. At the time point *j*, we now have a list of TFs and their influence score, *IL*(*t*), that are measured in the previous step. It, then, initialize the most influencing TF (i.e., argmax_*t*_ *IL*(*t*)) as a set of seed *S* and conducts network propagation on the TF network *G* to update *IP*(*v*). Then, it measures the similarity of ranking, SCC, between *IP*(*v*) and *DE*(*v*). It adds the next most influencing TF into the set of seed *S*, performs network propagation, computes SCC, and decides whether accepting the TF or not accepts; the TF is accepted if SCC increases or declined otherwise. It continues this process for the list of TFs until the coverage of target genes exceeds the half number of DEG at the time point. Finally, it produces *S* as a set of major regulatory TFs.

### 3.5. Data Description

The experiments for the evaluation of PropaNet were conducted using two time-series gene expression datasets measured under thermal stress, AtGenExpress (Kilian et al., 2007) and E-MTAB-375 (Caldana et al., 2011), and a TF network, PlantRegMap (Jin et al., 2016).

- **AtGenExpress dataset**. AtGenExpress (Kilian et al., 2007) dataset was a well curated dataset that measured time-series gene expression data for multiple treatments from multiple tissues of Arabidopsis, making it four-dimensional dataset (gene, time, condition, and tissue). In this analysis, the AtGenExpress data of thermal (cold and heat) stress from shoot tissues were used. The raw data, derived by AtGenExpress experiments, were downloaded from GEO (Barrett et al., 2013) for cold (GSE5621) and heat (GSE5628) stresses. Also, zero time point data were downloaded from control time-series samples (GSE5620). Among the dataset of heat stress in AtGenExpress, recovery phase data were excluded to focus on the response of stress. Then, the data were processed using justRMA function of affy R library (Wagner, 2016) with default options (background and RMA normalization). To handle multiple probes in a single gene, custom CDF (Dai et al., 2005) was also used as input of justRMA to summarize expression levels for gene IDs. DEGs of each time point were determined by limma (Ritchie et al., 2015) package using replicates. In this way, the gene expression dataset of 14 samples (7 time points including 0 time point × 2 replicates) for cold stress and 10 samples (5 time points including 0 time point × 2 replicates) for heat stress and the corresponding DEG profiles were generated. The detailed information of the AtGenExpress samples was summarized in Supplementary Table 1.
- **E-MTAB-375 dataset**. A dataset E-MTAB-375 (Caldana et al., 2011) of light condition with 22 time points under cold and heat stress was used. Since E-MTAB-375 provided no replicates at each time point, we used the first and the second time point data (i.e., *T* = 0 and 5 min) of non-stress time-series for the replicate data at zero time point (i.e., *T* = 0 min). Accordingly, the 5-min time point data of stress (cold/heat) time-series were discarded for consistency of time point. The raw data of E-MTAB-375 were downloaded from ArrayExpress (Kolesnikov et al., 2014) and processed in the same way as the AtGenExpress data were processed. The detailed information of the E-MTAB-375 samples was summarized in Supplementary Table 2.
- **PlantRegMap TF network**. PlantRegMap (Jin et al., 2016) (http://plantregmap.cbi.pku.edu.cn) was used as a template network in our analysis. PlantRegMap was a TF network that was constructed mainly from TF ChIP-seq data. Other existing TF networks, such as ATTED-II (Obayashi et al., 2017), AraNet (Lee et al., 2014), and ATRM (Jin et al., 2015), were constructed by integrating multiple information such as TF ChIP-seq data, literature, and co-expression information. Since we were not able to evaluate how accurate each of the networks is, we decided to use PlantRegMap since we thought that PlantRegMap was the most unbiased network mainly form TF ChIP-seq data. After the PlantRegMap network including 688 TFs and 192,385 regulatory relations was downloaded, we computed PCC between the TF-TG pairs from the experimental gene expression data and assigned PCC values to the weights of edges of the template network.

### 3.6. Experiment Procedures for Evaluation of PropaNet

To evaluate PropaNet, we performed four types of experiments as below.

- **Investigation on biological implications of time varying networks in response to thermal stress in Arabidopsis**. We demonstrated PropaNet by applying it to the AtGenExpress microarray dataset to investigate the response mechanism of thermal (cold and heat) stress in Arabidopsis. The temperature-related stress has been investigated extensively in scientific, agricultural, and industrial fields because of recent climate and weather extremes, derived by global warming. Moreover, climate and weather extremes would be worse as global warming continues; a special report of Intergovernmental Panel on Climate Change (IPCC) in 2018 predicts with high confidence that global warming is likely to reach 1.5°C between 2,030 and 2,052 if it continues to increase at the current rate (IPCC, 2018).
  Using the AtGenExpress dataset and the PlantRegMap (Jin et al., 2016) template network, PropaNet generated time varying networks in response to thermal stress. Then, enrichment analysis was conducted for the Gene Ontology (GO) (Consortium, 2018) terms and the Kyoto Encyclopedia of Genes and Genomes (KEGG) (Kanehisa and Goto, 2000) pathways to investigate the biological function of the time varying networks by performing Fisher's exact test with Benjamini-Hochberg correction (Benjamini and Hochberg, 1995) using statsmodels (Seabold and Perktold, 2010) Python library. In addition, PropaNet TF networks were compared to a literature-based network, ATRM (Jin et al., 2016), a literature-based network including 1,432 TF-TG edges, to investigate how many TF-TG relationships in the predicted network were reported in the literature.
- **Performance comparison with existing tools**. This experiment quantitatively compared PropaNet with existing methods at TF and gene levels. For the TF level comparison, the PCC, SCC, entropy and DREM (Schulz et al., 2012) methods predicted the stress-related TFs by analyzing the AtGenExpress dataset, and the accuracy of prediction of each tool was measured in terms of *F*_1_ score by comparing the predicted TFs to ground truth TFs. For the gene level comparison, existing methods to determine stress-responsive genes from time-series data, EDISA (Supper et al., 2007), OPTricluster (Tchagang et al., 2012), and DREM (Schulz et al., 2012), were compared with PropaNet by running them on the AtGenExpress dataset and comparing the resulting genes to the ground truth genes in the same way as TF level comparison analysis did.
  To define the ground truth genes, a list of genes that were annotated with cold/heat response-related GO terms, such as “response to cold,” “cold acclimation,” and “cellular response to cold” for cold stress, and “response to heat,” “heat acclimation,” “cellular response to heat,” and “cellular heat acclimation” for heat stress was collected from the TAIR database (Lamesch et al., 2012). Then, 33 and 20 TFs and 330 and 158 genes were collected as ground truth genes for cold and heat stress, respectively.
  The precision, recall, and *F*_1_ scores were used as accuracy measures to investigate how many ground truth genes in the predicted networks, which were defined as follows:
  $$\begin{array}{lll} \text{precision} & = & TP/(TP+FP)\\ \text{recall} & = & TP/(TP+FN)\\ F_{1} & = & \left(\frac{{\text{precision}}^{-1}+{\text{recall}}^{-1}}{2}\right)\\ = & 2\cdot \frac{\text{precision}\cdot \text{recall}}{\text{precision}+\text{recall}}, \end{array}$$
  where *TP*, *FP*, and *FN* were the number of correctly positive-predicted genes, falsely positive-predicted genes, and falsely negative-predicted genes, respectively.
- **The effect of utilizing non-stress time-series sample as control**. Many biological experiments were designed to compare treated vs. control samples. Use of control samples can be used to eliminate the effects of background biological mechanisms (e.g., circadian rhythm) and help focus on mechanisms related to treated samples. To investigate the effect of utilizing control sample data, we developed a modified version of PropaNet, “PropaNet^C^,” to utilize non-stress time-series control sample data for generating time-varying networks.
  After processing stress and non-stress time-series data, PropaNet^C^ identified DEGs at each time point compared to the initial time point (0 h) for each of control and treated samples separately. PropaNet^C^ then selected seed DEGs at each time point, *t*, by subtracting the DEGs of control samples from DEGs of stress samples (i.e., $DEGset_{t}^{stress}-DEGset_{t}^{control}$, where “−” was set difference operation). Using the seed DEGs, PropaNet^C^ conducted further steps, influence maximization and network propagation, in the same way as PropaNet did. We performed analysis with and without using control samples, i.e., PropaNet and PropaNet^C^, on the AtGenExpress dataset and compared the resulting TF networks.
- **Analysis of the effect of the number of time points**. In this experiment, we investigated how PropaNet handled the dataset with many time points. The goal of the experiment was to compare two datasets with different time points as below:
  – Cold stress data
    - AtGenExpress, 7 time points between 0 and 24 h from shoots
    - E-MTAB-375, 22 time points between 0 and 21 h 20 min under low-light condition
  – Heat stress data
    - AtGenExpress, 5 time points between 0 and 3 h from shoots
    - E-MTAB-375, 22 time points between 0 and 21 h 20 min under normal-light condition

To investigate the effects of the number of time points and the length of time intervals, we compared how many genes were overlapped between two adjacent time points. The reason for this criterion was to investigate on the effect of the densely measured transcriptome data in the time domain for the construction of time varying TF networks. The overlap of two adjacent networks was defined as the number of gene sets common between two adjacent time points. Quantitatively, the overlap was defined as $\frac{A\cap B}{A\cup B}$, where *A* and *B* gene sets at two adjacent time points.

## 4. Results

### 4.1. Investigation on Biological Implications of Time Varying Networks in Response to Thermal Stress in Arabidopsis

**Figure 2:**
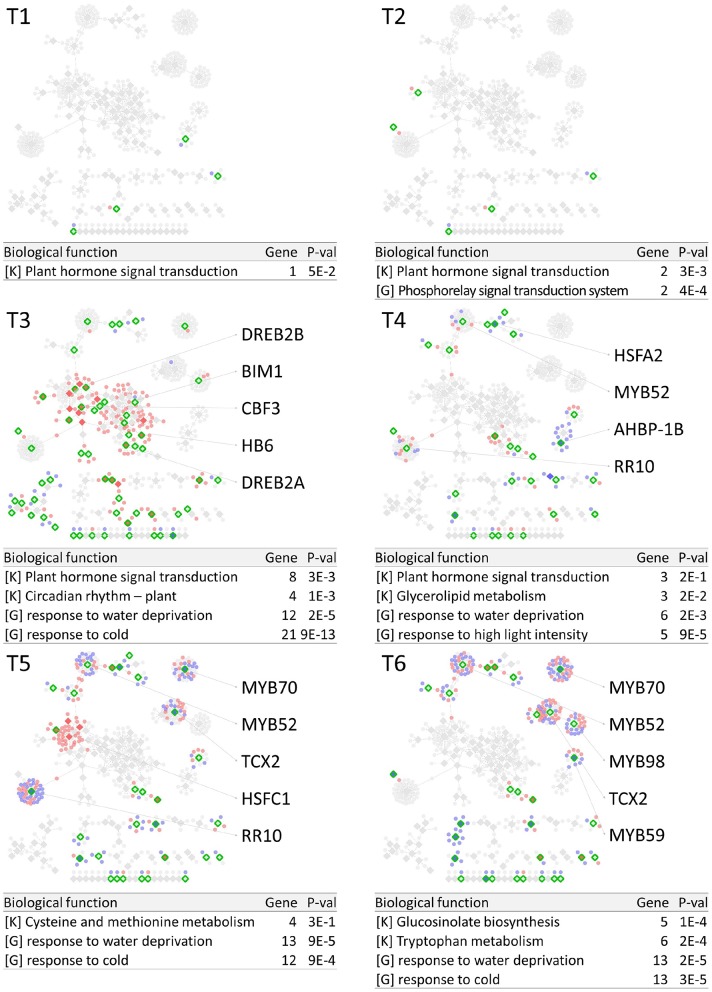
Time-varying network of six time points (T1 ~ T6) for cold stress experiment data in Arabidopsis. Red and blue nodes represent up/down DEGs, and green border rhombuses represent the identified regulatory TFs. Tables show top-2 enriched terms for each of KEGG pathways [K] and GO terms [G] with adjusted *p*-values by Benjamini-Hochberg correction. Some interesting TFs are named in the figure.

**Figure 3:**
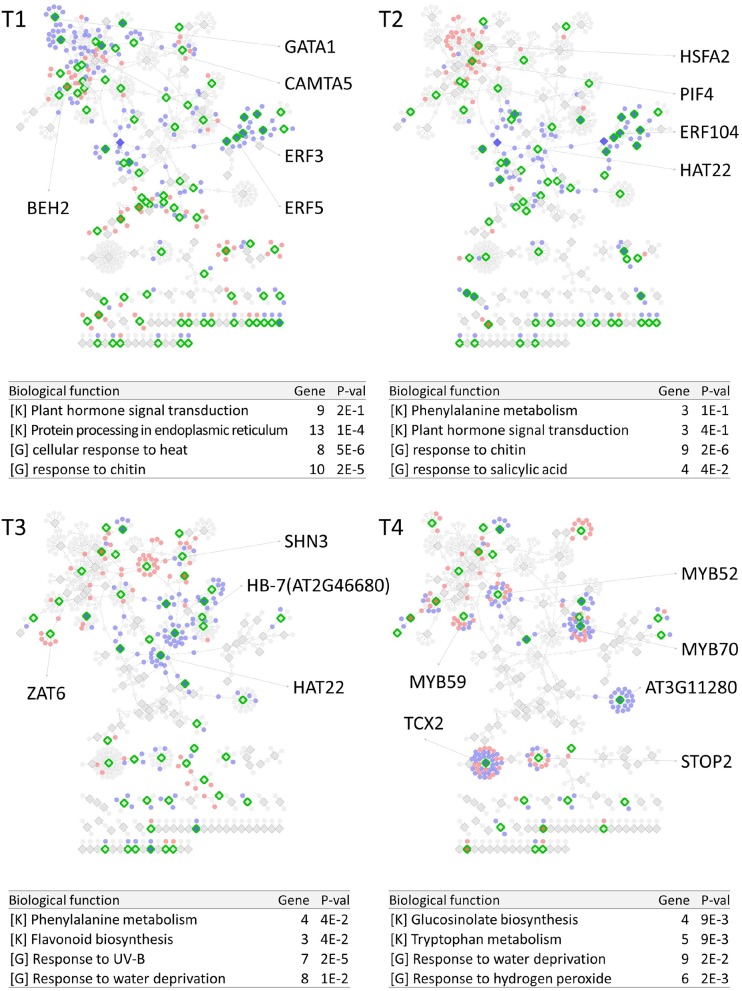
Time-varying network of four time points (T1 ~ T4) for heat stress experiment data in Arabidopsis. Red and blue nodes represent up/down DEGs, and green border rhombuses represent the identified regulatory TFs. Tables show top-2 enriched terms for each of KEGG pathways [K] and GO terms [G] with adjusted *p*-values by Benjamini-Hochberg correction. Some interesting TFs are named in the figure.

PropaNet investigated time-varying TF networks using time-series gene expression data measured at seven (*j* = 0, …, 6) and five (*j* = 0, …, 4) time points for cold and heat stresses, respectively. DEGs were detected at each of the six time points (*j* = 1, …, 6) and four time points (*j* = 1, …, 4) for cold and heat stresses, DEGs were detected at each time point with respect to zero time point (*j* = 0). The number of DEGs were 41, 23, 524, 2,262, 6,129, 7,656 for cold stress, and 1,177, 522, 1,915 and 7,424 for heat stress. Then, PropaNet produced six-time-point-networks (*j* = 1, …, 6) for cold stress (Supplementary Table 3) and four-time-point-networks (*j* = 1, …, 4) for heat stress (Supplementary Table 4). Figures 2, 3 show (1) the visualization of time varying TF networks for each time point, (2) interesting TFs that have relatively many downstream genes in the network, and (3) the enriched GO terms and pathways of the target genes, for the analyses of cold and heat stress data networks.

The time-varying networks had a modular structure of genes, i.e., clusters of genes were spread out throughout the network. In addition, neighboring modules had causal relationship where a module activated at the previous time point cause the activation of neighboring modules. This trend showed propagation of TFs to other genes over time under cold and heat stress. An interesting observation is that the cold stress network showed more delayed response than other types of stress in terms of the number of DEGs on the time domain, which was also reported in an earlier study on AtGenExpress dataset (Wanke et al., 2010a). Most genes in cold stress network show little response in the early time point (T1 and T2), but stress response starts from T3 time point in cold stress network while the heat stress network showed response at the very first time point, T1. In addition, major regulatory TFs that have many target genes in the network were well-known TFs related to each stress. For example, CBF3 was found in the T3 cold stress network, which is the most well-known TF for the response to cold stress that initiates global gene expression change to the cold stress (Medina et al., 2011). In addition, cold stress is known to be closely related to drought and heat stresses. Cold, and osmotic stresses are known to induce expression of many of the same genes and downstream genes in Arabidopsis (Xiong et al., 2002). In our result, the drought stress-responsive TFs such as DREB2A and DREB2B appeared in the early response stage (T3) cold stress network. Heat shock factors such as HSFA2 and HSFC1 appeared in T3 and T4 cold stress networks, and they are documented to be induced in cold stress to regulate downstream heat-shock-related proteins (Swindell et al., 2007). In heat stress network, HSFA2 (Schramm et al., 2006), ERF family TFs (Mizoi et al., 2012) that are known to regulate the expression level of downstream genes in heat stress appeared in the heat stress network.

The GO and KEGG enrichment analysis of cold stress networks showed the “response to cold” and “response to water” GO terms and the “plant hormone signal transduction” KEGG pathway were enriched, which are known to be related to cold stress in the literature (Eremina et al., 2016). For heat stress analysis, the “response to chitin” GO term and the “hormone signal transduction” KEGG pathway were enriched, and these terms are known to be related to heat stress in the literature (Eremina et al., 2016).

To investigate how many TF-TG relationships in the PropaNet network were reported through the previous experiments, the PropaNet network was compared to a literature-based network, ATRM (Jin et al., 2016). Among 580 and 687 edges of the PropaNet networks for cold and heat stress, 80 and 64 edges were literature-supported. The overlap between the edges of PropaNet network and the edges of ATRM network was statistically significant (*p* < 10^−7^ and *p* < 0.0098 by Fisher's exact test for cold and heat stress networks). Among the literature-supported edges, 7 edges were supported by the literatures of cold-specific experiments, such as DREB2A→LTI78 (Xiong et al., 2001), DREB2A→LTI30 (Chung and Parish, 2008), CBF3→ERD10 (Seki et al., 2002), HOS10→LTI78, HOS10→COR15A, HOS10→NATA1, and HOS10→ADH1 (Zhu et al., 2005). Also, 10 edges of the PropaNet heat stress network were supported by the literatures of heat-specific, such as HSFA9→HSP101 (Kotak et al., 2007), WRKY39→MBF1C (Li et al., 2010), DREB2A→HSP70 (Sakuma et al., 2006), HSFA2→APX2 (Charng et al., 2007), HSF1→HSP17.6A (Nishizawa et al., 2006), HSF1→GolS1, HSF1→GolS2, HSF1→MIPS2, HSFA2→GolS1, and HSFA2→GolS2 (Busch et al., 2005).

### 4.2. Performance Comparison With Existing Tools

**Figure 4:**
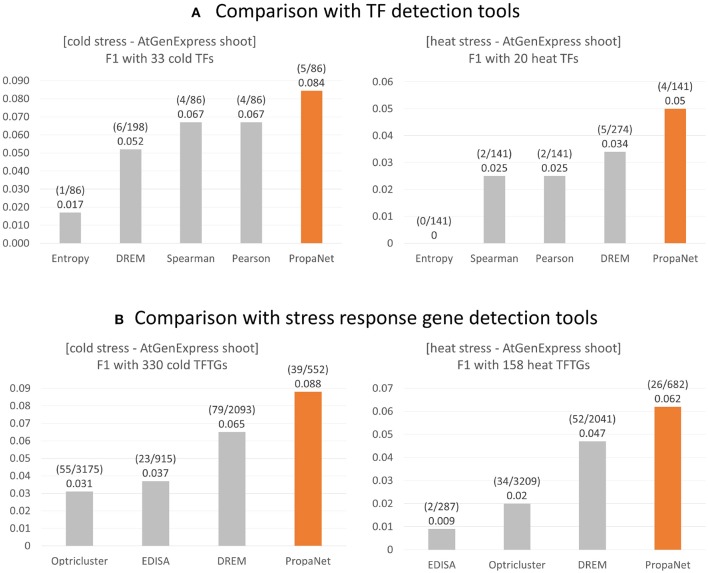
Performance comparison of stress response gene detection tools. **(A)** For major TF selection, PropaNet was compared with PCC, SCC, entropy, and DREM in terms of detecting how many ground truth TFs, TFs that were annotated by cold/heat stress GO terms. **(B)** For regulatory network construction, PropaNet was compared with EDISA, OPTricluster, DREM in terms of detecting how many ground truth stress response genes that were annotated by cold/heat stress GO terms. *F*_1_ score was used to measure the accuracy of tools.

The performance comparison was done at TF and gene level using ground truth genes. For the TF level comparison, PropaNet was compared with the PCC, SCC, entropy and DREM (Schulz et al., 2012) methods, and PropaNet performed best for both cold and heat AtGenExpress datasets (Figure 4A). The precision, recall, and *F*_1_ scores of the TF level comparison were summarized in Supplementary Table 5.

For the gene level comparison, existing methods to determine stress-responsive genes from time-series data, EDISA (Supper et al., 2007), OPTricluster (Tchagang et al., 2012), and DREM (Schulz et al., 2012), were compared with PropaNet. PropaNet showed the best performances for both cold and heat stress datasets (Figure 4B). The precision, recall, and *F*_1_ scores of the gene level comparison were summarized in Supplementary Table 6.

### 4.3. Effects of Utilizing Non-stress Time-Series Sample as Control

**Figure 5:**
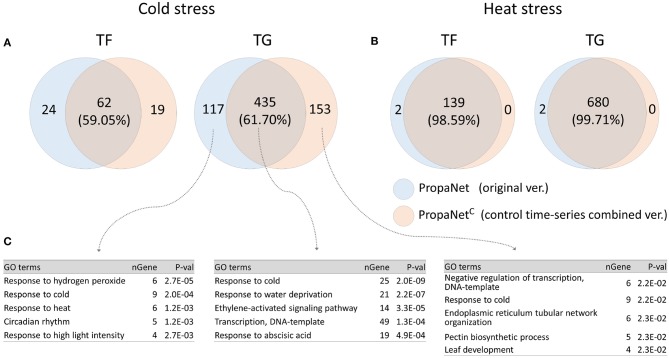
Effects of using non-stress time-series control samples. PropaNet^C^ was a modified version that utilized non-stress time-series control data. The Venn diagram showed that PropaNet and PropaNet^C^ produced about 60% overlapped results in cold stress **(A)** over 98% overlapped results in heat stress **(B)**. **(C)** Enriched GO terms in target genes for cold stress showed that a list of circadian rhythm genes were excluded in the analysis result by PropaNet^C^ (*p* < 10^−8^).

PropaNet^C^ is a modified version of PropaNet to utilize non-stress time-series control sample data. We performed analysis using PropaNet and PropaNet^C^ on the AtGenExpress datasets and investigated how much the resulting networks were overlapped. About 60% of analysis results in cold stress and over 98% in heat stress were overlapped (Figures 5A,B). The GO enrichment analysis for cold stress showed that the cold stress-related GO term, “response to cold,” was enriched in analysis results from PropaNet and PropaNet^C^ overlapping genes(*p* < 10^−8^). The GO term, “circadian rhythm,” was enriched only in the results from PropaNet-specific genes (*p* < 0.0012) (Figure 5C). In detail, circadian rhythm-related genes, such as *LNK3, ERD7, WNK1, CCR2*, and *GI*, were not enriched in the analysis result from PropaNet^C^. This observation suggests that the use of non-stress time-series control sample data could eliminate the effects of background biological mechanisms, e.g., circadian rhythm from the network analysis result.

### 4.4. Effects of the Number of Time-Points

**Figure 6:**
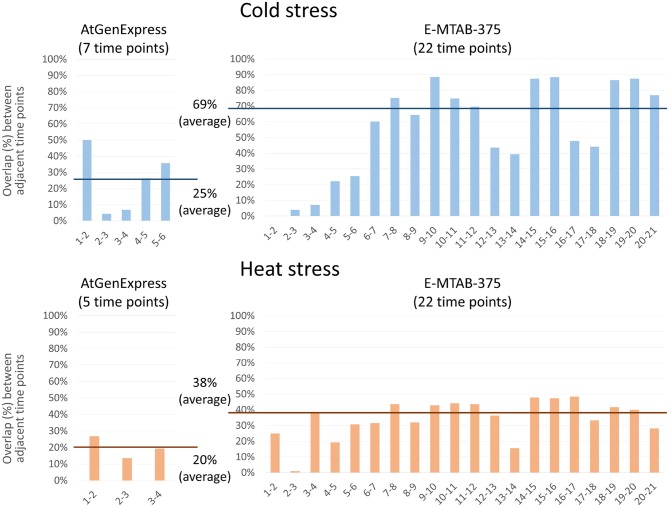
Effect of the number of time-points. PropaNet performed network construction analysis using AtGenExpress (nTimePoints = 7 and 5, for cold and heat stress) and E-MTAB-375 datasets (nTimePoints = 22, for both cold and heat stress). Then overlap between the results of adjacent time points were measured. The results of many time points (E-MTAB-375) showed more overlap between adjacent time points, 69% and 38% in average, than the results of small time points (AtGenExpress), 25% and 20% in average, for cold and heat stress, respectively.

We performed PropaNet analysis on two datasets with different time points, AtGenExpress (7 and 5 time points for cold and heat stress) and E-MTAB-375 (22 time points). We then investigated the overlap between the resulting networks of adjacent time points. Genes in the networks of many (densely sampled) time points (E-MTAB-375 dataset) were overlapped more, 69% and 38% in average, between adjacent time points than the resulting genes of small (loosely sampled) time points (AtGenExpress dataset), 25% and 20% in average, for cold and heat stress, respectively (Figure 6). This result is reasonable and shows the possibility of estimating gene expression values at unobserved time points as shown in our previous work (Kang et al., 2019).

## 5. Discussions

### 5.1. PropaNet Results and Stress Response Genes

The effects of temperature on plants are broad. The characteristics of effects of temperature on plant growth could be classified by imposed severity, duration, the ramp rates of changes, recovering condition and the developmental stages of the plant. Usually, the ambient temperature is not a stressful treatment. However, it may depend on the duration of exposure. The physiological consequences of sudden temperature treatment have been extensively studied. However, most of the experimental designs focused on experimentally available conditions and tissues such as leaves, roots, and fruits. Scrutinizing overall relationship of genes provides many of the unrevealed contexts of signal transfers and hierarchies among transcription factors.

In samples curated in AtGenExpress dataset (Kilian et al., 2007), the growth conditions of the pre-treated are different in terms of the treated periods. The plants grew under long-day conditions such as 16 h light and 8 h dark at a light intensity of 150 umol photons flux per square meter per second during the pre-treated stage. However, the photon flux was changed in the cold room to 60 umol in the same unit at steady state up to 24 h. It is our understanding that the circadian rhythm which maintained during the pre-treated stage might be interfered when the cold treatment started, and plants were exposed to the lower intensity light along with the cold temperature. Therefore, it is reasonable to accept that genes were affected by changes in light condition and circadian rhythm as well as cold stress. To remove the effect of diurnal cycling, light signaling and light dependent development, we compared the results of PropaNet with and without using non-stressed control samples. The major TFs described in the following sections are TFs that were detected even after removing the effect of diurnal cycle and light signaling.

#### 5.1.1. Biological Implications of Major TFs Detected From Cold Stress Data

A first glance at the results of cold stress at T1 stage, the appearance of AMS (ABORTED MICROSPORES) gene is matching with the previous reviews of temperature sensing mechanism of plants (Ma et al., 2012). AMS like TF has edges to genes like CYP81D11, UGT78D2, and AT4G00040. CYP81D11 might function as a monooxygenase, and its expression pattern suggests that it is involved in plant detoxification processes (Köster et al., 2012). UGT78D2 glycosylates the hydroxyl group at C3 to form cyanidin3-O-glucosides and plays a role in quercetin and kaempferol glycosylation in Arabidopsis (Li et al., 2017). AT4G00040 encodes chalcone and stilbene synthase family protein and interacts with AMS that binds to the promoter region of the putative CHS gene (Xu et al., 2010). It is noteworthy that bZIP proteins such as bZIP60, AHBP-1B and an HDZip (homeodomain-leucine zipper) protein HB5, are detected in early stages of cold stress T1 and T2. These bZIPs are known to be related to the response to stress as follows. The expression of bZIP60 is upregulated by ER stress inducers (Zhang et al., 2017), and AHBP-1B is responsible to a pathogen (Sun et al., 2018). A HDzip protein HB5 is a positive regulator of ABA pathway (Perotti et al., 2017). In addition, RGA1 that is a GRAS family TF regulates one of the histone deacetylase complex subunits. It may be involved in the nucleosome stability and possibly has a function as a transcription regulator (Zheng et al., 2018a). Those genes are relatively short-lived but have functioned as the early regulators although we could not identify the successive link to the signal cascade.

At T3 stage, major TFs of the networks are bHLH proteins (PIF4, MYC2), AP2/ERF proteins (RAP2.12, DREB1A, DREB2A, DREB2B, ERF9), heat shock protein (HSFA2), HOS10 and ZFHD1. It is remarkable that PIF4 which is known as a thermosensing TF is detected in our analysis. The expression of PIF4 is known to be gradually decreased at night under the light/dark transition (Nusinow et al., 2011). However, PIF4 gene expression seems to maintain stable status without fluctuation up until 6h after cold stress started under the low light intensity and then tend to decrease slowly in AtGenExpress data. PIFs are known to interact with other bHLH proteins including MYC2 and light and thermosensing genes such as Cry1 and PhyB (Park et al., 2018a; Xu et al., 2018). Our analysis, however, detected other potential targets genes such as RLP31, CYCD1, TPPH and AT1G19000 and these relationships remained even after the varying diurnal expression trajectories of the light signaling components were subtracted. This result suggests other unknown functions of PIF4 and their target genes in stress responses besides light and thermosensing. APETALA2/Ethylene Responsive Factor (AP2/ERF) family TFs are related to biotic and abiotic stress regulation in plants (Zhang et al., 2018). Among the AP2/ERF proteins found by PropaNet, DREB1A was also detected as a cold stress-specific marker gene in terms of the transcript abundance and fold change (Wanke et al., 2010a). DREB2A is known as a key transcriptional activator that induces many heat- and drought-responsive genes in Arabidopsis (Mizoi et al., 2019). HSFA2 is a well-studied thermo-responsive gene. In our analysis, HSFA2 interacted with heat shock factors like GolS1, GolS2, HSP101, MBF1C, and ELIP1. When the control samples are considered, however, the network of HSFA2 and their target genes was detected only at the last time point, which suggests that these genes may react to light changes in control samples during the middle stages of cold stress, but the effect may be removed from the last time point network. HOS10 negatively regulates COR gene that acts downstream of the CBF proteins and might control ABA-mediated cold acclimation (Janmohammadi and Mahfoozi, 2013). ZFHD1 belongs to a family that binds to the promoter region of the early responsive to dehydration stress 1 (ERD1) gene and upregulates several stress-inducible genes (Elfving et al., 2011).

In the late stage of cold stress, MYB genes (MYB52, MYB70, MYB98, MYB59) act as central TFs of signaling cascades. MYB52 is known to be involved in ABA, drought, salt, and cold responses (Yu et al., 2016). MYB59 is shown to be involved in cell cycle regulation and presumed to control K-specific negative regulation of primary root elongation in Arabidopsis (Nishida et al., 2017). MYB59 targets several kinases and phosphatases, therefore it is reasonable to guess it could regulate such activities. MYB70 is reported that it negatively regulates genes related to developmental, hormonal and stress signaling pathways (Barah et al., 2015). In our analysis, MYB70 targets many of histone-related genes including histone H2A that takes the position of the variant H2A.Z. MYB98 is a specific transcription factor in synergid and regulates the expression of the female attractant LURE1 (Li et al., 2015). MYB98 targets TCX2 (Tesmin/ TSO1 like CXC 2) which is a metal binding protein. TCX2 is a member of seven homologs of TSO1 and shows highly similar expression patterns compared to TSO1 (Sijacic et al., 2011) which forms a TSO1-MYB3R1 module that regulates cell proliferation with differentiation and is involved in floral organ differentiation, meristem regulation, and gametophyte development. TSO1/LIN54 to MYB3R1/ B-MYB regulatory module in plants and animals, which plays a critical role in coordinating the cell cycle with the cell fate commitment (Sijacic et al., 2011; Wang et al., 2018).

As shown in the list of major TFs, a number of TFs belong to a few protein families such as bZIP, AP2/ERF, heat shock proteins and MYB families. The major TFs of the same family can be detected together because they have common target genes or they are interaction partners for each other composing a dimmers or multimers. The interactions between TFs in the latter case have also been detected in an existing network analysis of AtGenExpress dataset (Wanke et al., 2010b).

#### 5.1.2. Biological Implications of Major TFs Detected From Heat Stress Data

In the first stage, our analysis revealed that many of AP2/ERF and hormone-related genes such as ERF2, ERF3, ERF38, RAP2.10, GATA1 are responsible to heat stress. ERFs form a representative TF family, AP2/ERF, that plays a role in development and stress response process of plants (Vogel et al., 2014). GATA is a transcription regulator, subdivided into four classes, and they are known to be involved in the control of greening, plant development, GA metabolism, and a lot of biological processes (Behringer and Schwechheimer, 2015). Contrast with cold stress, during the high-temperature treatment, WRKY57 is the TF at the initial response even though AMS like TF also is detected in our analysis at the first stage of temperature response. Unlike with cold stress, most plants grow well under higher ambient temperature. Higher ambient temperature is accelerating growth and differentiation. In some case, some organs in G0 stage of cell cycle turn on cell division process, resulting in the additional growth and re-differentiation.

In the following stages, HSFA2, MYB28 and ZAT6 are detected as major TFs. HSFA2 is detected as a major TF in both cold and heat stress data analysis. However, the target genes of HSFA2 are different under cold and heat stress. During the heat treatment, HSFA2 shows a relationship with partly common (HSP101, MBF1C, ELIP1, GolS1, and GolS2) and different sets (SIP2 and APX2) of gene comparing with cold treatment. SIP2 is presumed to be involved in phloem unloading of raffinose in sink leaves. Raffinose and other members of the raffinose family oligosaccharides are involved in stress tolerance and act as antioxidants (Clauw et al., 2016). APX2 encodes cytosolic ROS scavenging enzyme and is shown to be activated in response to heat stress in an HSFA3-dependent manner (Katano et al., 2018). In addition, an early study of AtGenExpress dataset found HSFA2 as a heat stress-specific marker gene by its transcript abundance and fold change (Wanke et al., 2010a). MYB28 is a remarkable major TF which interacts with CYP83A1, CYP83B1, MTO1, and SUR1. The CYP family is made up of a wide variety of monooxygenases containing a prosthetic heme group, and CYP83A1 is required for aliphatic glucosinolate biosynthesis (Nagano et al., 2014). CYP83B1 catalyzes the first committed step in the indole glucosinolate sub-pathway, converting indole-3-acetaldoxime to an S-alkyl-thiohydroximate adduct (Maharjan et al., 2014). MTO1 and CGS1 construct the carbon/amino skeleton derived from Asp with the sulfur moiety donated by Cys (Hacham et al., 2017). SUR1 encodes the C-S lyase that functions in indole glucosinolates biosynthesis. SUR1 is important to IAOx-dependent IAA synthesis (Kong et al., 2014). ZAT6, zinc finger of Arabidopsis thaliana 6, was also found as one of the plant core environmental stress response genes of Arabidopsis responsive to diverse stress conditions, by its dynamic gene expression trajectory (Hahn et al., 2013).

#### 5.1.3. Dynamic Changes of Major TFs and Their Functions in Cold and Heat Stress

We observed dynamic changes of TF-TG grouping and their biological functions in the time course analysis of cold and heat stress data as shown in Figures 2, 3, respectively. Under the cold condition, we can see only a few TF genes that had a signal at the initial T1 and T2 stages. Major grouping occurs in T3 stage, which suggests the defense system related stress may be turned on at this stage, showing AP2/ERF proteins (RAP2.12, DREB2A, DREB2B, ERF9) as major TFs in our analysis. The TF-TG group of T3 stage propagates to the outside of the network in the following T4, T5 and T6 stages to regulate cell and organ restructuring against hazardous effects of cold temperature. About 5–6 TF-TG groups in the later time points have such major TFs like MYB52, 59, 70, 98, and TCX2. It seems that TF-TG network changes in order to prepare for whole-plant-wide harmful cold stress. These changes are focusing on osmotic defense mechanism and structural reforming. However, under the higher temperature condition, the initial responses of the plant are merged into hormone metabolism. We could not observe that critical changes during T1 and T2 stages. In our analysis, as strong growth control compounds, such as gibberellin, ethylene and auxin-related TFs like AP2/ERF and GATA, were detected in early stages. This growth and reforming stages turn to defensive stages in T3 and T4. Many of the stress-related TFs like MYB, TCX2, and ZAT6 were observed as major TFs in T3 and T4 stages under heat stress.

### 5.2. Advantages and Limitations of PropaNet

Comparative analysis results showed that PropaNet detected known stress-responsive genes more accurately than other time series analysis methods (see section 4.2). The advanced performance of PropaNet can be explained with its three characteristics distinguished from other methods. First, PropaNet considers indirect regulatory power of a TF though consideration of multiple steps of transcription regulation (by influence maximization) while existing tools consider direct targets only. Second, PropaNet takes into account of the regulatory power of multiple TFs simultaneously (by network propagation). Third, PropaNet uses “differential expression” values (such as z-scores or log_2_ fold changes) to prioritize TFs that target more significant DEGs.

We conjecture that the consideration of multiple steps of regulation of a TF and simultaneous regulations of multiple TFs is the main reason why PropaNet performed better than existing tools in terms of F1 scores. Additionally, the use of PropaNet^C^ that considers non-treated control samples in PropaNet analysis showed to reduce the effect of background biological mechanisms (e.g., circadian rhythm) on time-varying networks (see section 4.3). Therefore, it is recommended to use PropaNet^C^ to eliminate the effects of background biological mechanisms when both treated case and non-treated control samples are available.

On the other hand, there are a few limitations of PropaNet that have to be considered before analysis. PropaNet assumes the reliability of a template TF network that is given by the user as input. In our experiment, we used PlantRegMap as a template network, which was generated by identifying TF binding sites using TF ChIP-seq and searching the DNA sequence motif on the promoter regions of the target genes. Then, it appended TF-target interactions that were found in the literature. Thus, it has the possibility to include false TF-target interactions; (1) The antibodies that are used in TF ChIP experiments are known to have the range of affinity to bind the un-targeted but similar-structured TFs. (2) The quality of ChIP-seq data can vary depending on the experimenters and the year of data generation. (3) TF ChIP-seq experiments are conducted in a particular condition, so it is possible that the TF interactions change in a different condition. Use of extended versions of PlantRegMap or protein-protein interaction networks may provide more detailed information on the plant response to cold and heat stress, which has to be studied in the future. In addition, PropaNet detects TFs of which differential expressions are positively correlated with DEGs, which cannot capture inhibitory relationship between TF and target genes. This is due to the limitation of random walk process that might not converge with negative weights. Thus, incorporating negative weights to detect inhibitory TFs can be a future work for PropaNet as well.

## Data Availability

All datasets for this study are included in the manuscript and the Supplementary Files.

## Author Contributions

SK and WJ designed the project. SK, KJ, and HA directed the development of the algorithm. SK and KJ developed a modified version of influence maximization algorithm. HA collected and processed the data. KJ, DJ, MP, and HA implemented the program of the algorithm and conducted experiments of network construction. WJ and JH biologically interpreted the network analysis results. HA, KJ, SK, and WJ wrote the paper.

### Conflict of Interest Statement

The authors declare that the research was conducted in the absence of any commercial or financial relationships that could be construed as a potential conflict of interest.
